# Exploring how patients respond to GP recommendations for mental health treatment: an analysis of communication in primary care consultations

**DOI:** 10.3399/bjgpopen19X101670

**Published:** 2019-10-30

**Authors:** Joseph Ford, Felicity Thomas, Richard Byng, Rose McCabe

**Affiliations:** 1 Postdoctoral Research Associate, College of Medicine and Health, University of Exeter Medical School, St Luke’s Campus, Exeter, UK; 2 Senior Research Fellow, College of Medicine and Health, University of Exeter Medical School, St Luke’s Campus, Exeter, UK; 3 Professor of Primary Care Research, Community and Primary Care Research Group, University of Plymouth, Faculty of Medicine and Dentistry, Plymouth Science Park, Plymouth, UK; 4 Professor of Clinical Communication, School of Health Sciences, City, University of London, London, UK

**Keywords:** mental health, social sciences, research methods (other), patients, primary health care, general practice

## Abstract

**Background:**

Patient take-up and adherence to antidepressants and talking therapy is low. However, little is known about how GPs recommend these treatments and whether patients accept them.

**Aim:**

To examine how GPs recommend antidepressants and talking therapy, and how patients respond.

**Design & setting:**

A total of 52 recorded primary care consultations for depression, anxiety, and stress were analysed.

**Method:**

Using a standardised coding scheme, five ways doctors recommend treatment were coded, conveying varying authority and endorsement. The treatment recommendation types were as follows: more directive pronouncements (I’ll start you on X); proposals (How about we start X?); less directive suggestions (Would you like to try X?); offers (Do you want me to give you X?); and assertions (There are medications that might help). It was also coded whether patients accepted, passively resisted (for example, withholding response), or actively resisted (for example, I’ve tried that before).

**Results:**

A total of 33 recommendations occurred in 23 consultations. In two-thirds of cases, GPs treated the patient as primary decision-maker by using suggestions, offers, or assertions. In one-third of cases, they used more directive pronouncements or proposals. GPs endorsed treatment moderately (67%), weakly (18%), or strongly (15%). Only one-quarter of recommendations were accepted immediately. Patients cited fears about medication side effects and/or dependency, group therapy, and doubts about treatment efficacy. Despite three-quarters of patients resisting, 76% got prescriptions or self-referral information for talking therapy.

**Conclusion:**

Initially, GPs treat patients as the decision-maker. However, although patients resist, most end up with treatment. This may impact negatively on treatment uptake and success. Social prescribing may fill a treatment gap for some patients.

## How this fits in

Research has indicated low patient adherence for antidepressants and low uptake rates for counselling. Using a standardised coding scheme to analyse how GPs recommend treatment and how patients respond in recorded real-life consultations, this article shows that GPs overwhelmingly leave decisions about these treatments to patients. It also suggests that patients frequently resist such recommendations, sometimes explaining their reasons for doing so (concerns about dependence, efficacy, and so on). Patients who resisted typically ended up being given the treatments anyway. Analysis of cases where patients resist suggests discussing and providing information about treatment options before making a recommendation could improve patient acceptance and uptake.

## Introduction

Mental health conditions account for a significant proportion of primary care consultations: around one-third of GP visits in the UK are prompted wholly or partially by mental health concerns.^1^ The National Institute for Health and Care Excellence (NICE) guidelines for the treatment of common mental health disorders (depression and anxiety) recommend antidepressants and talking therapy, either separately or in combination for more severe cases.^2^ In 2016, 64.7 million prescriptions for antidepressants were issued in England, which was a 108.5% increase since 2006.^3,4^ However, despite this accumulation, most new courses of antidepressants are not completed.^5^ In 2017–2018, there were also 1.44 million new talking therapy referrals, which is a 3.9% increase on 2016–2017.^6^

There has been intense debate about the efficacy of antidepressants for mild to moderate depression,^7,8^ and there is a range of views within the profession on whether antidepressants are being under- or overprescribed.^9,10^ There are similar questions about the efficacy of antidepressants for anxiety disorders.^11^ These issues are of practical relevance to GPs, who may wish to offer patients the hope that comes with offering treatment while also having doubts as to the efficacy of that treatment.^12^

People diagnosed with depression and anxiety also express wide-ranging views and preferences on the benefits and problems associated with antidepressant use.^13–15^ After being prescribed antidepressants, less than one-fifth of patients took antidepressants in line with clinical guidelines over 6 months.^5^ Moreover, 31% of talking therapy referrals ended before treatment began.^6^ Despite the controversial nature of antidepressant treatment in primary care, little is known about how decisions to start antidepressants and initiate patient referral to talking therapy are negotiated and endorsed in GP–patient consultations.

Doctor–patient communication about treatment beyond mental health has been the focus of recent research in primary^16^ and secondary^17,18^ care. By analysing recordings of doctor–patient communication, this work has shed light on who is driving treatment decisions: of particular interest here is how strongly the doctor recommends the treatment; whether the GP or the patient is the primary decision-maker; and how positively the patient responds to the possibility of starting treatment.

These issues are reflected in subtle differences in how GPs formulate the recommendation; for example, ‘I’m going to start you on an antidepressant’ versus ‘Would you be interested in taking an antidepressant?’ and whether patients actively accept or resist the recommendation. These formulations range from highly directive, where the doctor makes the decision without soliciting the patient’s input, to non-directive, where the doctor leaves the decision to the patient.^16^ Interestingly, they also appear to reflect the doctor’s perspective on the perceived clinical benefit for the patient through stronger or weaker endorsement of the treatment.^16^

Similarly, subtle differences in how patients respond to treatment recommendations reflect acceptance or resistance and hence their stance on starting the treatment in question. A number of studies on acceptance and resistance in interaction have shown that acceptance occurs quickly and positively. Meanwhile, resistance can be passive or active: passive resistance involves a delayed response, a withheld response, a minimal verbal response (for example, mhm) or non-verbal response (a nod). Active resistance includes statements such as ‘I don’t want to take any medication’ or questioning the recommendation.^19–21^

Understanding how GPs and patients negotiate antidepressants and talking therapy on an interactional level offers a detailed view of what actually happens in practice. This can shed light on patient response to starting treatment in the consultation and also the wider social debate about treatment for depression. Hence, the aims of this study were to analyse how GPs recommend and endorse antidepressants and talking therapy in routine consultations in primary care, and whether patients accept or resist these recommendations.

## Method

### Data and sampling

The data for this study were taken from the One in a Million archive^22^ of recorded primary consultations and analysed as part of a wider study (the DeStress project) on poverty and mental health.^12^ These recordings were collected from 23 GPs in 12 practices in the west of England between July 2014 and April 2015. Patients were approached before their appointments with participating GPs and, if they agreed to take part, their consultation was recorded. In total, 421 patients were approached, 21% (*n* = 87) of whom declined to participate in the study. Of the 334 who did consent to take part, seven recordings failed. See Jepson *et al* ^23^ for further information on the One in a Million study.

Three hundred patients consented for their recording to be used in research. These 300 consultations were screened for common mental health problems, that is, anxiety, depression, and stress (excluding conditions that were not mood related, such as psychosis and memory problems), using their International Classification of Primary Care, Second edition (ICPC-2) codes (see *Appendix 1* in supplementary materials for list). This left a total of 52 recordings for inclusion in this study, 48 of which were video, four of which were audio.

### Treatment recommendations

The recordings were examined for sequences in which doctors recommended antidepressants or that the patient refer themselves to talking therapy. These two forms of treatment are different in that medication involves a decision that is made and acted on by the doctor within the consultation (that is, giving a prescription) while talking therapy requires the patient to take further action after the consultation by calling the number that the doctor has given them. For talking therapy, this is standard practice as psychology services advocate patients taking the step to make the initial call themselves.

Each sequence was then transcribed in detail to illustrate characteristics of speech such as pauses, overlap, stress, intonation, and pace using Jeffersonian conventions (see *Appendix 2* for a glossary).^24^These characteristics, in particular pauses and overlap, are important in communicating acceptance of or resistance to a recommendation.

Each recommendation was coded using the Treatment Recommendation Coding Scheme,^25^ which was developed in US and UK primary care. This scheme codes for the first treatment recommendation in the data. While there may have been prior reference to treatment, this is the point at which a decision about treatment becomes relevant. The coding scheme considers both the type of recommendation that the GP uses and the level of endorsement, that is, how strongly they recommend the particular treatment. A small number of cases were coded in group sessions with the research term before the full body of cases was coded by two of the authors. Cohen’s kappa (κ) was used to calculate inter-rater reliability.

In accordance with the protocol, the GPs’ recommendations for antidepressants or talking therapy were coded as one of the five recommendation types:

1. Pronouncements — patients are given no choice; for example, ‘I will start you on medication.’.
2. Proposals — patients are invited to support or collaborate with the doctor’s idea; for example, ‘How about we try medication?’.
3. Suggestions — patients are given the choice; for example, ‘Would you like to try medication?’.
4. Offers — doctors show a willingness to prescribe for the patient without actively supporting the treatment; for example, ‘Do you want me to give you medication?’.
5. Assertions — doctors state that treatment exists without suggesting it is good for the patient; for example, ‘There is a medication available.’.

The GP treatment recommendation was further categorised as: (1) doctor as decision-maker, that is pronouncements or proposals, which situate the doctor as the primary decision-maker; or (2) patient as decision-maker, that is, suggestions, offers, or assertions, which leave the patient to decide.

Doctor endorsement was also coded as weak (for example, ‘It would be worth reconsidering starting an antidepressant’), moderate (for example, ‘So maybe some talking therapy would be good’), or strong (for example, ‘I’m gonna give the you the contact details’) based on modal construction of the offer. Endorsement was based on the strength with which a GP used a particular type of recommendation. As noted above, endorsement may be a reflection of the GP’s perspective on the likely clinical benefit that the treatment has for the patient.

### Patient responses

The patient response was coded as: (1) acceptance: quick positive acceptance, for example, ‘I’d like to have that’; or (2) resistance: passive resistance was indicated by a minimal verbal or non-verbal acknowledgement, for example, ‘mhm’, nodding, or no response; or active resistance was indicated by, for example, questioning the purpose of medication or indicating a wish not to take the medication; for example, ‘I’m not very keen, I don’t want to take more tablets.’

## Results

In total, there were 33 treatment recommendations involving 13 GPs across 23 consultations. Of these consultations, 16 were with female patients, and seven were with male patients. At the time of the consultation, 14 of these patients were already receiving some form of treatment so the recommendation was either for an additional form of treatment or an adjustment to existing treatment. The mean patient age was 46 years (standard deviation [SD] 18, range 19–84 years). The shortest consultation was 7:13 minutes, and the longest consultation was 25:51 minutes (mean duration 15:28 minutes, SD 0.21). Of the recommendations, nine were recommendations to start medication (27%), five to review an existing medication (15%), and 19 to start talking therapy (58%).

### How do doctors recommend and endorse antidepressants and talking therapy to patients?

Almost two-thirds (60%) of GPs’ recommendations treated the patient as the primary decision-maker: 18% using suggestions, 24% using offers, and 18% using assertions. In only 39% of cases did GPs deliver their recommendations in a way that treated the GP as the primary decision-maker: 15% using pronouncements, and 24% using proposals. Cohen’s kappa suggested strong agreement between coders (κ = 0.959, *P*<0.001).

#### Patient as decision-maker

An example of how GPs position the patient as the decision-maker can be seen in Box 1, lines 1 and 3: the GP suggests that the patient consider talking therapy: 'So maybe some cognitive behavioural therapy would be good?' This marks this as their opinion while leaving open the possibility that the patient may have their own ideas about the best course of action. The patient accepts and does indeed leave the consultation with information about cognitive behavioural therapy. As above, the transcripts are all presented using the Jeffersonian^24^ transcription conventions.

Box 1 **[Case 14: 09:17]**

01   Doc: M::m. Yeah. .thhh [S- Yea:h. **So may:be: some] cognitive**=

02   Pat:                 [(                       )]

03   Doc: =**behavioural therapy: [would be go]od.** Because tha- you

04   Pat:                       [Yea:h.      ]

05   Doc: hav- (0.3) you haven’t had that befor:[e and it’s] a slightly=

06   Pat:                                 [No.     ]

07   Doc: =approach.

#### Doctor as decision-maker

By comparison, in Box 2 the GP, at lines 1–2, uses a directive pronouncement: 'I’m gonna give you a contact detail for something called [talking therapy service].' The decision has already been made without eliciting the patient’s perspective on this course of action. As can be seen in line 3, there is no opportunity for the patient to respond and display their perspective on this course of action. They respond minimally, in overlap with the GP. They subsequently leaves the consultation with information about therapy ( see Box 3 for additional examples of each treatment recommendation type.)

Box 2 **[Case 32: 13:17/18:34]**

01  Doc: But there’s more .hhhh the- y’know so the- **I’m gonna give**

02        **you a contact detail for something called [Service]?**

03        [Which i:s     ] (0.5) (*eh eh*) it’s- it’s: psychological=

04  Pat:  [((Nods)) Okay. ]

05  Doc: therapies.=So [  talk]ing therapies.

06  Pat:         [Okay. ]

Box 3 **Examples of treatment recommendations and responses**

Example 1: Pronouncement

*In this consultation, the patient wishes to restart their antidepressants, having tried unsuccessfully to wean themselves off them. In the course of talking about this, the GP also takes the opportunity to recommend talking treatment.*

01  Doc:   If you fee:l they’re not worki:ng or: .hh u::m y’know things are

02     getting worse then obviously come back and we [can- we] can=

03  Pat:                          [Yea:h.  ]

04  Doc:   =think about what else to do, [.hhhh] **U::m: and I’ll give you=**

05  Pat:                 [Okay.]

06  Doc:   **=the number for ((Ser[vice)),** .hhh A:n:d see what they say,=

07  Pat:              [(((Nods))

08  Doc:   =[And if that] doesn’t wo:rk but you want (.) private

09  Pat:    [M::m:.    ]

10  Doc:   =counselli:ng then u:m pt. .hhh there’s: a (0.4) g- a s- u::h

11     service called ((Name)) Serv[ice? .hhh    ] And they have=

12  Pat:               [Oh ri:ght. Yea:h. ]

13  Doc:   =a l:i:st of all the accredited (.) the:rapists: in the area,

Example 2: Suggestion

*In this consultation, the patient has recently stopped their antidepressants. Their mood has worsened as a result, and they have come to the GP about this. As the extract begins, the GP is raising the possibility of restarting an antidepressant.*

01  Doc:   Also: .hhhh u::m give:n (0.5) th- all your symptoms:: it sounds

02     like that y’know that: you::r depression has quite- has really

03     dipped. N’okay¿ .hhh (.) You’re now not taking any (.) you’ve

04     taken yourself off the citalopram and that’s been some

05     time.=It’s been July:. .hhh **So:: it:: (0.7) (eh) and (eh) (0.4)**

06     **it would be: (ww)worth considering restarting an antidepressant.**

07     .hhh Maybe an alternative antidepress- You could go back on

08     Citalopram, Okay¿ Which will help with the anxiety and will help

09     with the depression,=And you did tolerate it well in the

10     past.=That’s grea- You didn’t have side effects on it.

11     [.hhhh] No. That would be one option.

12  Pat:   [No.  ]

Example 3: Proposal

*In this consultation, the patient has recently started antidepressants. While these have had some benefit, they have also had various side effects. The extract begins as the GP is recommending that the patient stay on their current dose for the time being.*

01  Doc:   BUT U::M for some rea:so:n we find that fifteen miligra:ms

02     .hhh at night ti:me .hhh when (we started it) can be quite

03     sedating at night time [but it ] helps people with sleep.=

04  Pat:            [Right.]

06  Doc:   =.hhhh **I** **would propose (0.3) (eh) (0.3) NOT to increa- because**

07     **you’ve only been on it (y’know) [just** **] under two weeks okay.**

08  Pat:                 [Okay.]

09  **Doc:** **.hhh To continue a:s is for another two weeks,** [I’ll    ] review=

10  Pat:                        [Yeah.]

11  Doc:   =you in two weeks’ time,

Example 4: Offer 

*In this consultation, the patient has experienced a flare-up of their bulimia, having recently moved to the UK to study. The GP has recommended various courses of action that they can take and, as the extract begins, is recommending a psychological service for people with eating disorders.*

01  Doc:   And the other thing that might be helpful is that we have (0.4)

02     u:m an eating disorders servi:ce called ((Service)), U::m pt. .hh

03     which is tck. for people- mainly for people with bulimia

04     actuall[y:,   ] **Um and I could refer you to them,**

05  Pat:     [Yea:h.] I would really

06     app[reciate that.]

07  Doc:   [    U::m] and they would (.) they would see: you for

08     <psychological input> side of things.

Example 5: Assertion

*In this consultation, the patient suffers from various physical health issues (about which they have spoken to the GP before) that have, in turn, impacted on their mental health (about which they have not spoken to the GP before). As the extract begins, the GP is describing the treatments that are available for depression.*

01  Doc:   I mean I think in- in broad ter:ms I would say there are

02     three things that are- are (.) are- are quite helpful in

03     depression. Okay. .hhh **Um .hh (0.4) one of them is- is (.)**

04     **counselling,**

05     (0.3)

06  Pat:   ((Nodding)) Ye[ah.]

07  Doc:           [Whi]ch comes in different sor:ts and

08     flavours, Okay.

#### Levels of endorsement

In line with their non-directive approach, GPs rarely offered strong endorsement for medication or talking therapy. The most common level of GP endorsement was moderate (67%), followed by weak (18%), and then strong (15%). Cohen’s kappa suggested strong agreement between coders (κ = 1.000, *P*<0.001).

An example of weak endorsement can be seen in Box 3, example 2: ‘It would be worth considering restarting an antidepressant.’ The key features marking this as weak are the modal construction ‘it would be’ and the suggestion that the patient considers restarting an antidepressant. An example of moderate endorsement, meanwhile, can be seen in Box 3: ‘So maybe some talking therapy would be good.’ Here, the doctor again uses a modal construction ‘would be good’; although without the additional mitigating features that made the previous example weak. An example of strong endorsement, finally, can be seen in Box 2 ‘I’m gonna give you a contact detail for something called [Service].’ What makes this a strong endorsement is that the GP states, unconditionally and without mitigation, what they are ‘going’ to do.

### How do patients respond to these recommendations?

#### Acceptance

Patients initially accepted GPs’ recommendations in only one-quarter (24%) of cases. Examples of two such cases can be seen in Box 1 and Box 2: ‘Yeah’ (line 4); and ‘Okay’ (line 4). The patients in these examples make it clear that they are agreeing to the treatments that their GPs are recommending. They do so, furthermore, without delay (and, in Box 1, in overlap with the recommendation itself).

#### Resistance

In almost three-quarters (67%) of cases, patients resisted treatment recommendations, either passively (73%) or actively (27%). When actively resisting, patients expressed: a general aversion to medication (see Box 4); fears about dependency on medication; concerns about attending a group session (for therapy); and doubts about the efficacy of treatment (see Box 5). In 67% of the active resistance cases, the patient’s concern was addressed quickly, either by abandoning the recommendation entirely (50%) or addressing the concern (33%). In 17% of cases, however, the patient continued to express concern even after the doctor’s initial attempts to address it, leading to extended, 4-minute-long negotiation sequences (see, for example, Box 5). Cohen’s kappa suggested strong agreement between coders (κ = 0.758, *P*<0.001).

Box 4 **[Case 17: 14:09/21:39]**

01  Pat:   And I tri:es to put a brave face on it ↑don't ↑↑I.

02  Com:  Yeah.

03      (0.4)

04  Com:  (Yeah/You) try to keep going but (0.6)

05  Pat:   .shih

06      (0.6)

07  Pat:   It gets HARd~e::r.~

08  Com:  it's getting increasingly difficult.

09       (1.8)

10  Doc:  **D’you think it might be a good idea: to have somethi:ng to pick**

11      **you u:p a bi:t with your moo:d.**

12      (2.2)

13  Pat:   Well I could t- sh- (.) try something.=I mean I'm not one to

14      take a loada tablets anyway ( ) [but__  ] .hhh=

15  Doc:                 [Mm-hm.]

16  Pat:   =could have somethi:ng to hhhh (0.6) .hhh ↑to pick me up I↑

17      ↑s’po:s::e.

18      (0.9)

19  Pat:   .shih

20  Doc:  Or maybe a bit of counselli:ng.

21      ((Patient sniffling)) (2.7)

22  Doc:  ‘Cos you've bee:n through a lo:t haven't you¿

23      (1.8)

Box 5 **[Case 01: 04:07/18:07]**

01  Doc:   Would you think about seei:ng the mental health

02     tea:m again.

03     (2.4)

04  Pat:   No::h. Well (0.6) no. Because I:: um (2.2) hh I can’t uh- I

05     don’t think it would help.

06  Doc:   What about trying something to (.) try: and make your

07     mood a bit better.

08     (1.2)

09  Pat:   I don’t really want any more- any medication either.

10     ((4 minutes, 45 seconds omitted--discussion about patient’s

11     physical health))

12  Doc:   But do you think maybe talking to someone about tha:t might- I

13     know it might be very very difficult, .hhh But y’know ((patient

14     name)) you could live for many many more years and you’re

15     obviously not happy as it is now, .hh Although y:-

16     (0.6) I can take on board that you’re not keen for

17     a medication or talking to someone what if (.)

18     something like tha:t actually did help?

19     (0.3)

20  Doc:   E:ven if it was difficult initially.

21     (2.0)

22  Pat:   pt. .hhh (0.3) I don’t really think it wou:ld. I

23     think um:: pt. [.hh ]

24  Doc:        [But] how would you kn(h)ow £if you haven’t tried

25     it.£

26     ((2 minutes, 30 seconds omitted))

27  Doc:   So what are we going to do::.

28  Pat:   ((Doctor’s name)) you’re wonderful.

29     (0.9)

30  Pat:   I’m sorry whoever’s listening to this rubbish.

31     (1.1)

32  Doc:   I’m [not wonder]ful,

33  Pat:     [(From) me. ]

34     (0.4)

35  Doc:   I d- I- (.) But I d- I do w- (m-) I want you to

36     [think about] what I’ve said.

37  Pat:   [hhhh    ]     Yes. I [will.]

38  Doc:                [ Ab]out maybe

39     talking to someone.

40  Pat:   I will.

41     ((1 minute, 10 seconds omitted))

42  Doc:   Can I give you the number for this counselling in case

43     you do think you want to give it a g#o.#

44     (1.1)

45  Doc:   You just have to ring them you’re not committing to

46     anyth#ing.#

47     (2.8)

48  Doc:   Would you appease me?

49     (0.7)

50  Doc:   With taking the number?

51     (1.0)

52  Doc:   I’m gonna write it down for you.

53     (0.6) ((Doc begins writing))

54  Pat:   (Alººright.ºº/Oh.) Thank you.

In Box 4, the patient actively resists medication. They starts out noting that they have previously taken a stoical stance towards a stomach condition but that this has become ‘harder’ recently. In response to this, the GP recommends medication at lines 10–11 with a proposal (that is, a doctor’s idea for the patient to endorse): ‘D’you think it might be a good idea to try something to pick you up a bit with your mood.’ In line 12, there is a long 2.2 second delay. The patient then responds (lines 13–14, 16–17) with a disagreement marker ‘well’, followed by an account for why they would not like to take medication (they are ‘not one to take a load of tablets’, displaying a stoical stance) and partial agreement or partial disagreement, all of which mark resistance in conversation.

Indeed, the GP interprets the patient’s response as resistance. In response, the GP abandons this recommendation and proposes an alternative form of treatment (line 20): ‘Or maybe a bit of counselling.’

However, patient resistance did not always lead to the doctor abandoning their recommendation: in 76% of the cases where patients initially resisted GPs’ recommendations, they were ultimately given an antidepressant prescription, details for accessing talking therapy, or agreed to the doctor’s recommendations to adjust the patient’s medication dosage. Box 5 shows an example of a case where the patient goes from resisting to ultimately accepting the treatment.

Box 5 begins as the GP is recommending talking therapy for the patient’s long-term mental health issues. Previously in the consultation, the patient has stated that they do not want talking therapy or medication.

The GP’s treatment recommendation at lines 06–07 is a proposal with moderate endorsement: ‘What about trying something to try and make your mood a bit better.’ Following this, at line 08, there is a 1.2 second silence and no response from the patient. This delay and lack of patient uptake displays passive resistance.

There follows a discussion about the patient’s physical health, after which the GP in line 12, reiterates their earlier treatment recommendation: ‘What if something like that actually did help?’ The patient passively resists again by not responding (lines 19 and 21). The patient then voices their resistance more actively at lines 22–23 (they don’t think it would help), which the GP responds to with an objection of their own at lines 24–25: ‘But how would you know if you haven’t tried it.’

In contrast to the previous example, the GP has continued to pursue their treatment recommendation after the patient has resisted. This continues for several more minutes, with the GP raising the matter again after checking the patient’s blood pressure at line 27: ‘So what are we going to do.’ The patient responds by praising the doctor as ‘wonderful’ (line 28) and referring to themselves in a self-deprecating way (lines 30 and 33). The doctor disregards the patient’s compliment (‘I’m not wonderful’, line 32) but reiterates that they would like the patient to ‘think about what [they have] said.’ This is a downgraded form of their earlier recommendation, that is, ‘thinking about’ counselling as opposed to ‘trying’ it, which the patient agrees to at lines 37 and 40: ‘I will.’

To summarise, the patient has gone from resisting a recommendation to agreeing to ‘think’ about it. This lays the groundwork for later in the consultation, where they do indeed agree to take the information about the counselling. This starts at lines 42–43, with the GP offering to give the patient the number for counselling. Following a 1.1 second gap at line 44, the GP clarifies that there is no commitment associated with taking the number. The GP then, after another unanswered appeal (‘Would you appease me? ... With taking the number?’), says that they are ‘gonna write it down’ for the patient at line 52. The patient thanks them for doing so (line 54) and leaves the consultation with the phone number.

During the consultation, this patient has gone from resisting a recommendation for counselling to ultimately accepting the information about it. This highlights both the lack of acceptability of the GP’s original treatment recommendation and the additional work that was needed to solicit the patient’s acceptance to think about counselling. Indeed, it is notable that, while this GP started out with a type of recommendation that treated the patient as the primary decision-maker (a suggestion), they ultimately ended up using a type that situates themselves as the primary decision-maker (a pronouncement). It is also notable that their final appeal was based on their relationship with the patient (‘will you appease me?’) rather than the benefit that the treatment is likely to have. This indicates the delicate balance between facilitating patient autonomy, and GPs’ beliefs and interactional work to persuade patients to try talking therapy, if they believe this may benefit patients in the longer term,

A crosstabulation of acceptance and resistance by treatment type can be seen in Table 1.

**Table 1.** Cross-tabulation of treatment type, treatment recommendation, and patient response

**Treatment type**	**Patient response**	**Total**
Accepts	Resists
**Medication (start**)	2	7	9
**Medication (adjust**)	3	2	5
**Talking treatment (start**)	3	16	19
**Total**	**8**	**25**	**33**

## Discussion

### Summary

This micro-analysis of communication in primary care consultations shows that GPs overwhelmingly position the patient as the primary decision-maker when raising the possibility of starting antidepressants or talking therapy, and tend not to strongly endorse either treatment. Through the subtle ways in which they raise treatments, they mark the patient as decision-maker (suggestions, offers, and assertions) two-thirds of the time, and doctor as decision-maker (pronouncements and proposals) only one-third of the time. Meanwhile, patients tended to resist rather than accept antidepressants and talking therapy. Patients accepted treatment in only one-quarter of cases. They initially resisted treatment — passively or actively — in three-quarters of cases. Patients resisted treatment because of doubts about the efficacy of treatment based on previous experiences, fears about dependency and/or side effects, and concerns about attending group therapy. Despite patient resistance, 76% left the consultation with medication or information to self-refer to talking therapy.

It was noted above that, while GPs may wish to give patients hope for a treatment, this can be complicated by concerns about being too directive and, sometimes, their own doubts about the efficacy of treatment.^12^ The findings here support this, suggesting that, to a large degree, GPs offer only moderate endorsement of treatments and communicated with patients with common mental health problems as collaborative partners in the decision-making process. On a speculative note, it would be worth exploring in future studies whether low endorsement may engender resistance: if GPs do not convey strong support for a treatment, this may make patients more likely to reject those treatments. It would also be worth exploring whether more openness about effectiveness and medication side effects^26^ (and withdrawal) might result in higher adherence (albeit with lower numbers of prescriptions) and more efficient use of GP time. This would require more time than is currently available in primary care consultations.

When patients do resist, GPs often pursue acceptance of treatment. Analysis of one such case showed that while the GP started with a recommendation that situated the patient as the primary decision-maker, they increased their own involvement in, and endorsement of, the decision to achieve the patient’s acceptance, also appealing to their relationship with the patient to persuade the patient to think about talking therapy. There was thus a tension between the GP positioning the patient as the decision-maker initially while taking a more active role when the patient, in that role, resisted. This may be related to the time pressure in brief primary care consultations, where GPs wish to offer patients some options to move forward. It also echoes GP perspectives on working with people with mental health problems to guide them towards different understandings and ways of managing their problems over the longer term (Parker *et* *al*, unpublished data, 2019a).

### Strengths and limitations

The main strength of this study is that it used a standardised coding system^25^ that captures the subtle ways in which doctors recommend treatment and how patients respond. It has thus, for the first time, shed light on the process via which doctors and patients negotiate treatments for common mental health conditions. The use of a coding scheme does, however, to some extent involve decontextualising individual cases from the wider consultations in which they occurred. Hence, the nuances of individual cases could not be specifically analysed in this study.

A larger sample size would have enabled further comparisons between medication and talking therapy. Future research on this topic could use a more targeted form of data collection by sampling data on these criteria. It is also not known what patients did after leaving the consultation room. This means that in cases where patients were ultimately given information on self-referring to talking therapy, there is no way of knowing whether patients did indeed follow the GP’s recommendation.

### Comparison with existing literature

An earlier study on wider presenting problems in primary care using the same coding system^16^ found pronouncements were most common, accounting for 29% of cases. In the current study, pronouncements accounted for only 15% of cases. Other treatment recommendation types were broadly similar across both studies, suggesting that GPs are less likely to use directive types when it comes to treatment for common mental health problems.

Another study^27^ using this coding system found that patients in England tend to display resistance to both prescription and non-prescription medication, evincing a general scepticism towards medications. This article both supports and extends these findings, suggesting patients in England are not only sceptical about antidepressant medication but also that this scepticism extends to talking therapy. The main reason for patients’ scepticism noted in the earlier study was perceived inefficacy of treatments, which was also found in the current study.

These findings are also in line with the wider literature on treatments for common mental health problems. Interview studies, for example, have shown that patients starting and taking antidepressants frequently express concerns about side effects and dependency,^13–15^ with one study suggesting that patients value being able to discuss such issues with their GP (Parker *et al*, unpublished data, 2019b). Such concerns are reflected in these findings, both in the high levels of resistance and the reasons patients cite for resistance to these treatments. These findings could also be linked to the high levels of non-adherence for both antidepressants^5^ and counselling.^6^

### Implications for research and practice

When recommending mental health treatments, GPs overwhelmingly start by treating the patient as the primary decision-maker and do not strongly endorse available treatments. Although patients mostly resist treatment initially, most end up being given the treatment. If patients have not genuinely changed their mind, ongoing patient resistance is likely to lead to non-engagement in and lack of benefit from treatment. Awareness of, and discussion about, these concerns before making the recommendation could be of benefit to the patient and the doctor–patient relationship.

These findings suggest that the treatment options currently available in the UK, that is, medication and talking therapy, do not meet the needs of all patients. However, currently most GPs do not have other pathways or approaches to offer patients. Recognising that people’s health is determined by a range of social, economic, and environmental factors, there is increasing interest in addressing social causes of distress and social prescribing.^28^ Social prescribing involves GPs, nurses, and other primary care professionals referring people to a range of local, non-clinical services.

This could make a wider array of treatment options available for GPs to offer patients to address mental health problems.^29^ Currently, robust evidence on the effectiveness of social prescribing is limited, with most studies involving small-scale evaluations.^28^ The *NHS Long-Term Plan* specifically refers to more people benefiting from social prescribing and managing their health in partnership with the voluntary sector.^30^ However, availability of social prescribing is variable, largely beyond the GP’s control, and will need to be developed by policy-makers and local commissioners. Given patients’ resistance to existing treatment options, alternatives to medication and talking therapy that addresses social causes of distress could be more appealing for patients and GPs alike.
